# Efficient synthesis of furfurylamine from biomass *via* a hybrid strategy in an EaCl:Gly–water medium

**DOI:** 10.3389/fbioe.2023.1144787

**Published:** 2023-03-16

**Authors:** Wei He, Yu-Cai He, Jianren Ye

**Affiliations:** ^1^ College of Biology and the Environment, Nanjing Forestry University, Nanjing, China; ^2^ School of Pharmacy, Changzhou University, Changzhou, China; ^3^ College of Forestry, Nanjing Forestry University, Nanjing, China

**Keywords:** lignocellulose, furfural, furfurylamine, SO_4_^2−^/SnO_2_–HAP, deep eutectic solvent

## Abstract

The objective of this work was to develop an efficient approach for chemoenzymatically transforming biomass to furfurylamine by bridging chemocatalysis and biocatalysis in a deep eutectic solvent of EaCl:Gly–water. Using hydroxyapatite (HAP) as support, heterogeneous catalyst SO_4_^2−^/SnO_2_–HAP was synthesized for transforming lignocellulosic biomass into furfural using organic acid as a co-catalyst. The turnover frequency (TOF) was correlated with the pKa value of the used organic acid. Corncob was transformed by oxalic acid (pKa = 1.25) (0.4 wt%) plus SO_4_^2−^/SnO_2_–HAP (2.0 wt%) to produce furfural with a yield of 48.2% and a TOF of 6.33 h^-1^ in water. In deep eutectic solvent EaCl:Gly–water (1:2, v/v), co-catalysis with SO_4_^2−^/SnO_2_–HAP and oxalic acid was utilized to transform corncob, rice straw, reed leaf, and sugarcane bagasse for the production of furfural with the yield of 42.4%–59.3% (based on the xylan content) at 180°C after 10 min. The formed furfural could be efficiently aminated to furfurylamine with *E. coli* CCZU-XLS160 cells in the presence of NH_4_Cl (as an amine donor). As a result of the biological amination of furfural derived from corncob, rice straw, reed leaf, and sugarcane bagasse for 24 h, the yields of furfurylamine reached >99%, with a productivity of 0.31–0.43 g furfurylamine per g xylan. In EaCl:Gly–water, an efficient chemoenzymatic catalysis strategy was employed to valorize lignocellulosic biomass into valuable furan chemicals.

## 1 Introduction

Biorefinery concept is highly promising for the sustainable utilization of biomass to manufacture energy molecules, platform chemicals, and functional materials (Himmel et al., 2007; Delidovich et al., 2016; He et al., 2017a; Riva et al., 2021). The cost-efficient transformation of lignocellulosic biomass has attracted much attention for achieving a sustainable society (Cai et al., 2014; Guenic et al., 2015). Lignocellulose is mainly composed of three primary components including lignin, hemicellulose, and cellulose (Gong et al., 2019; Riva et al., 2021), which is regarded as a sustainable bioresource because of its availability, abundance, low cost, and renewable characteristics (Hu et al., 2014; Kaiprommarat et al., 2016). Hemicellulose, an amorphous heteropolysaccharide, is mainly composed of different C5 and C6 sugar monomers (Peleteiro et al., 2014; Lee and Wu, 2021). Xylan, which consists of *D*-xylose units, represents the main component of hemicellulose (Morais et al., 2020). As an important xylan-based product, furfural (FF) is a key building block (Zang and Chen, 2015; He et al., 2017b; Sweygers et al., 2018), which was listed in the “Top10 + 4” chemicals by the US Department of Energy (DOE) in 2004 (Bozell and Petersen, 2010). FF is a good solvent. It has been utilized as a renewable material for biofuels, detergents, lubricants, polymers, resins, food additives, pharmaceuticals (antiseptics and disinfectors), and agrochemicals (herbicides, insecticides, and pesticides) (Agirrezabal-Telleria et al., 2014; Douthwaite et al., 2017; Peng et al., 2019; Gong et al., 2022). It is an important precursor for widely synthesizing furan bio-based chemicals such as 2-methylfuran, furoic acid, cyclopentanone, furfuryl alcohol, succinic acid, furfurylamine (FAM), tetrahydrofuran, γ-valerolactone, and maleic acid (Lange et al., 2012; Möller and Schröder, 2013; Feng et al., 2016; Qiu et al., 2020; Zheng et al., 2020).

FAM, as a valuable furan-based chemical, has a vital role in manufacturing chemical intermediates, bioactive molecules, medicines, *etc.* (Dong et al., 2020). Industrially, FAM can be prepared *via* the chemical amination of FF under high temperature/pressure conditions, and this kind of amination requires a harsh performance and expensive catalysts, accompanied with potential environmental pollution (Zhang et al., 2019). Distinct from the chemical amination of FAM, biological amination of FF has gained great interest due to its mild performance condition, simple reaction, high catalytic activity, low toxicity, and environmental friendliness. ω-Transaminase is a pyridoxamine 5′-phosphate (PLP)-dependent enzyme, which has good selectivity for reversibly transforming the exchange of ketone groups (C=O) and an amino group (-NH_2_). The biomass-derived FF (90 mM) was aminated into FAM (74%) by *Escherichia coli* CV-PRSFDuet expressing ω-transaminase at 35°C (Zhang et al., 2019). To chemoenzymatically valorize biomass into FAM in a tandem reaction with a chemocatalyst and biocatalyst, it is necessary to employ an efficient strategy for the conversion of biomass into FF with biocompatible catalysts in an eco-friendly reaction system.

In recent years, homogeneous and heterogeneous catalysts have been utilized for FF production. Various acids (H_2_SO_4_, HCl, H_3_PO_4_, oxalic acid, formic acid, acetic acid, *etc*.) and salts (AlCl_3_, CoCl_2_, CrCl_3_, MaCl_2_, MnCl_2_, NaCl, NiCl_2_, SnCl_4_, *etc*.) are used as homogeneous catalysts (Rong et al., 2012; Enslow and Bell, 2015), which can be uniformly distributed in solvents. These homogeneous catalysts have high catalytic activity. However, their high loading will cause serious environmental pollution, and their recycle is a challenge. Heterogeneous catalysts that can be prepared by using a series of supports (e.g., graphene, zeolite, niobium oxide, niobium phosphate, sepiolite, kaoline, fly ash, carbon nanotube, resin, and carbon) have gained much attention because of their large surface areas, strong acidity, low corrosion, easy separation, and good thermal stability (Garcia-Sancho et al., 2013; Pholjaroen et al., 2013; Ma et al., 2019; Zhu et al., 2020; Di et al., 2021; Li et al., 2021). Although heterogeneous catalysts are easy to be recovered and have low corrosion (Li et al., 2019), the catalytic efficiency is still not satisfactory. The co-catalysis of homogeneous and heterogeneous catalysts might be used for the efficient transformation of biomass into FF, which deserves in-depth exploration.

In the production of FF from lignocellulosic biomass, used solvents have a crucial role in the enhancement of FF productivity (Lee and Wu, 2021). It is known that some organic solvents can confine the undesired side reactions (e.g., FF condensation and FF resinification) in water (Lam et al., 2012). Various solvents (e.g., dimethyl sulfoxide, ethanol, hexane, ionic liquids, toluene, γ-valerolactone, and dioctyl phthalate) have been employed to transform biomass or *D*-xylose, which resulted in the increased FF productivity (Tau et al., 2016; Xu et al., 2017; Widsten et al., 2018; Ma et al., 2020). Recently, it is of great interest to select more eco-friendly and thermostable solvents for enhancing FF production. Deep eutectic solvents (DESs), which are composed of mixing HBAs (hydrogen-bond acceptors) with HBDs (hydrogen-bond donors) (Li et al., 2018), have recently gained tremendous attention for FF production because of their low vapor pressure, good reusability, low toxicity, high thermostability, and ease of preparation (Xu et al., 2020; Pan et al., 2022). In acetone–ChCl:EG–water at 180°C, *D*-xylose was transformed to FF in 0.5 h at 75% yield by AlCl_3_ (Chen and Wan, 2019). Thus, DESs can be utilized as promising solvents for improving FF production because of their unique properties, which would restrict the undesired side reactions and promote FF formation.

**SCHEME 1:**
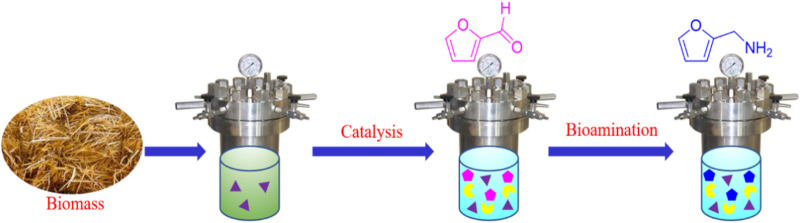
Chemoenzymatic conversion of biomass into furfurylamine (FAM).

To enhance FAM yield from renewable biomass, a chemoenzymatic conversion was developed in a tandem reaction by bridging chemocatalysis and biocatalysis in an eco-friendly DES–water system (Scheme 1). Hydroxyapatites (HAPs, Ca_10_(PO_4_)_6_(OH)_2_), which are naturally occurring phosphate minerals, were used as a carrier to prepare sulfonated tine-based heterogeneous catalyst SO_4_^2−^/SnO_2_–HAP. First, biomass was converted into FF *via* co-catalysis with a homogeneous catalyst and a heterogeneous catalyst in an EaCl:Gly–water system. An optimized system for the production of FF from lignocellulose was established by combined homogeneous organic acids with heterogeneous SO_4_^2−^/SnO_2_–HAP. The turnover frequency (TOF) was correlated with the pKa value of organic acids. The catalytic reaction parameters (e.g., type of organic acid, organic acid loading, SO_4_^2−^/SnO_2_–HAP loading, DES EaCl:Gly dosage, performance temperature, and catalytic time) were examined to improve FF production. Furthermore, biomass-valorized FF was biologically aminated into FAM in EaCl:Gly–water. An efficient chemoenzymatic strategy was employed to valorize biomass into valuable furan-based chemicals, realizing the high-value utilization of biomass.

## 2 Materials and methods

### 2.1 Reagents and materials

Corncob (CC), rice straw (RS), reed leaf (RL), and sugarcane bagasse (SB) were collected in a village market in Tieling city (Liaoning province, P.R. China). Hydroxyapatite (HAP), oxalic acid, SnCl_4_·5H_2_O, NaCl, and other chemicals were bought from Aladdin Industrial Inc. (Shanghai, China) and other commercial sources.

### 2.2 SO_4_^2−^/SnO_2_–HAP and DES EaCl:Gly preparation

Sulfonated heterogeneous catalyst SO_4_^2−^/SnO_2_–HAP was prepared with SnCl_4_·5H_2_O and HAP in a mass ratio of 1:3 in 1 L of ethanol by stirring. The preparation procedure of SO_4_^2−^/SnO_2_–HAP was carried out according to the method proposed by Zhang et al. (2019). DES EaCl:Gly was prepared according to the procedure proposed by Xu et al. (2020).

### 2.3 Co-catalysis of CC organic acids and SO_4_^2−^/SnO_2_–HAP in EaCl:Gly–water

To test the co-catalysis of biomass into FF with organic acids and SO_4_^2−^/SnO_2_–HAP on the effects of FF formation, water (40 mL), CC (7.5 wt%), and SO_4_^2−^/SnO_2_–HAP (2.0 wt%) were mixed with different pKa values of the organic acid (0.4 wt%) at 180°C for 10 min. For testing the oxalic acid dosage on the effect of FF generation, different loads of oxalic acid (0–0.6 wt%) were mixed with water (40 mL), CC (7.5 wt%), and SO_4_^2−^/SnO_2_–HAP (2.0 wt%) in a reactor (180°C) for 10 min. To establish an appropriate DES–water system for improving FF production, several volumetric ratios of EaCl:Gly–water (0:1, 1:3, 1:2, 1:1, 2:1, and 3:1, v/v) were examined in the transformation of CC (7.5 wt%) *via* co-catalysis with EaCl:Gly–water (40 mL), SO_4_^2−^/SnO_2_–HAP (2.0 wt%), and oxalic acid (0.4 wt%) in a reactor (180°C) for 10 min. To examine the performance temperature and reaction time of FF generation, co-catalysis with SO_4_^2−^/SnO_2_–HAP (2.0 wt%), EaCl:Gly–water (40 mL; EaCl:Gly–water volumetric ratio 1:2), and oxalic acid (0.4 wt%) in a reactor (160–180°C) for 5–40 min was carried out. After the given residence time at the certain performance temperature, this reactor was placed in an ice-water bath to cool down quickly. The product FF was determined using high-performance liquid chromatography (HPLC). FF yield is defined as follows:

$$\begin{array}{c} Yield\;of\;FF=\frac{FF\;produced\;\left(g\right)\times 0.88}{Xylan\;in\;biomass\;\left(g\right)}\times \frac{150}{96}\times 100\%. \end{array}$$

Molecular weights of *D*-xylose and FF are 150 and 96, respectively. The coefficient for catalyzing xylan in biomass into *D*-xylose is 0.88.

### 2.4 Bioamination of FF into FAM

The enzyme gene of ω-transaminase from *C. violaceum* ATCC 12472 (CV) was amplified. The linearized pET28aDuet-1 and purified CV were transformed into one *E. coli* DH5α and ligated by T5 exonuclease through an exonuclease-mediated seamless assembly. The obtained fragment was inserted into MCS-1 of pET28aDuet-1 after His-tag to obtain CV-pET28aDuet-1. *L*-Alanine dehydrogenase (AlaDH, GenBank ID 936557) from *B. subtilis* 168 was amplified. The fragments of CV and Ala were linked together *via* an overlap extension PCR. Ribosome binding sites were inserted between CV and AlaDH fragments. The ligated plasmid pET28a–CV–AlaDH was transformed into one *E. coli* BL21 (DE3). The constructed *E. coli* CCZU-XLS160 cells expressing CV ω-transaminase and AlaDH were cultured in a terrific broth by a supplement of ampicillin (100 mg/L) in a shaker (37°C; 180 rpm). When OD_600_ of CCZU-XLS160 cells reached 0.60, isopropyl β-*D*-1-thiogalactopyranosyl (IPTG) was added to the culture system at 20°C. After incubation for 20 h, CCZU-XLS160 cells were harvested for the biological amination of FF into FAM:

$$\begin{array}{c} Yield\;of\;FAM=\frac{FAM\;produced\;\left(mM\right)}{FF\;\left(mM\right)}\times 100\%. \end{array}$$

Biomass-derived FF, CCZU-XLS160 cells (0.05 g/mL), and NH_4_Cl (2 mol NH_4_Cl/mol FAL) were mixed in EaCl:Gly–water (1:2, v/v; pH 7.5) at 35°C. During the biological transamination, the samples were withdrawn. FF and FAM were quantified using HPLC.

### 2.5 Analytical methods

Components of CC were measured according to the procedure proposed by Selig et al. (2008). SO_4_^2−^/SnO_2_–HAP was captured using FT-IR, XRD, BET, and SEM procedures proposed by Peng et al. (2019). FF and FAM were quantified using Waters-2414 HPLC by using an Aminex HPX-87H column (Bio-Rad Laboratories, Hercules, CA) (Zhang et al., 2019). The mixture was composed of 80 vol% water, 20 vol% CH_3_OH, and 0.1 wt% trifluoroacetic acid, which was used as an eluent at a flow rate of 0.8 mL/min. FAM was determined at 210 nm, and FF was assayed at 254 nm.

## 3 Results and discussion

### 3.1 Characteristics of SO_4_^2−^/SnO_2_–HAP

**FIGURE 1:**
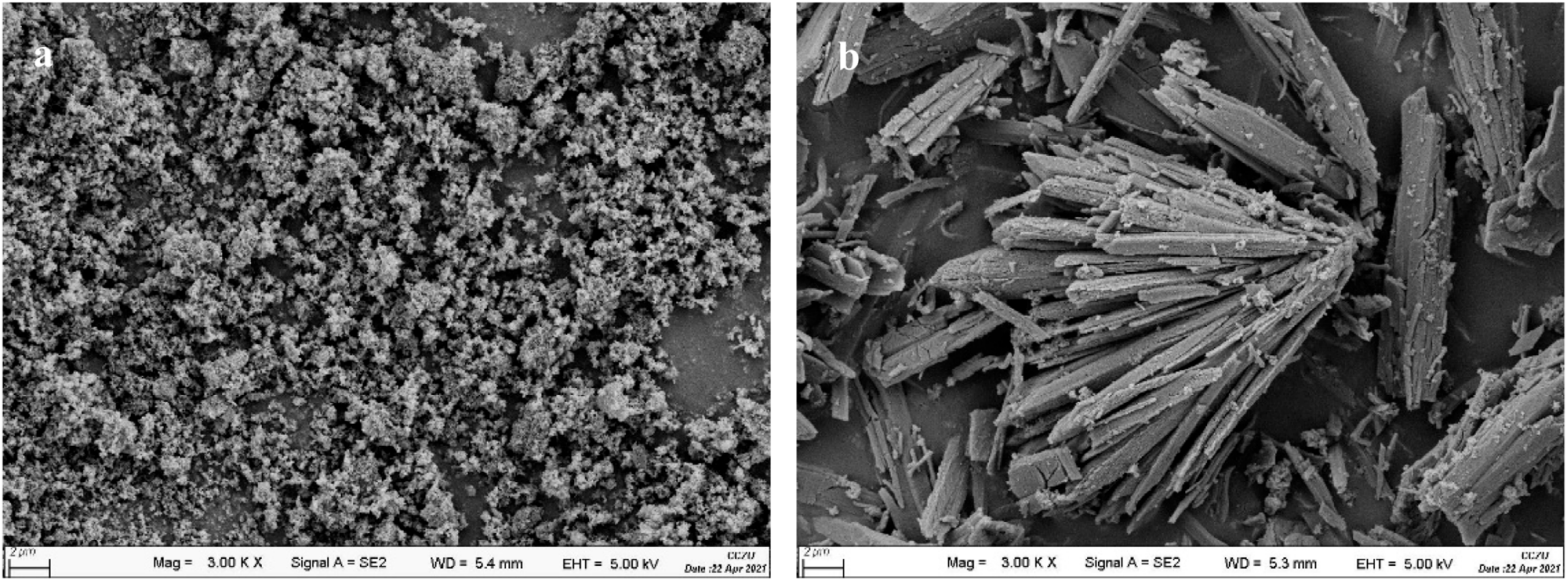
SEM of HAP **(A)** and SO_4_^2−^/SnO_2_–HAP **(B)**.

**FIGURE 2:**
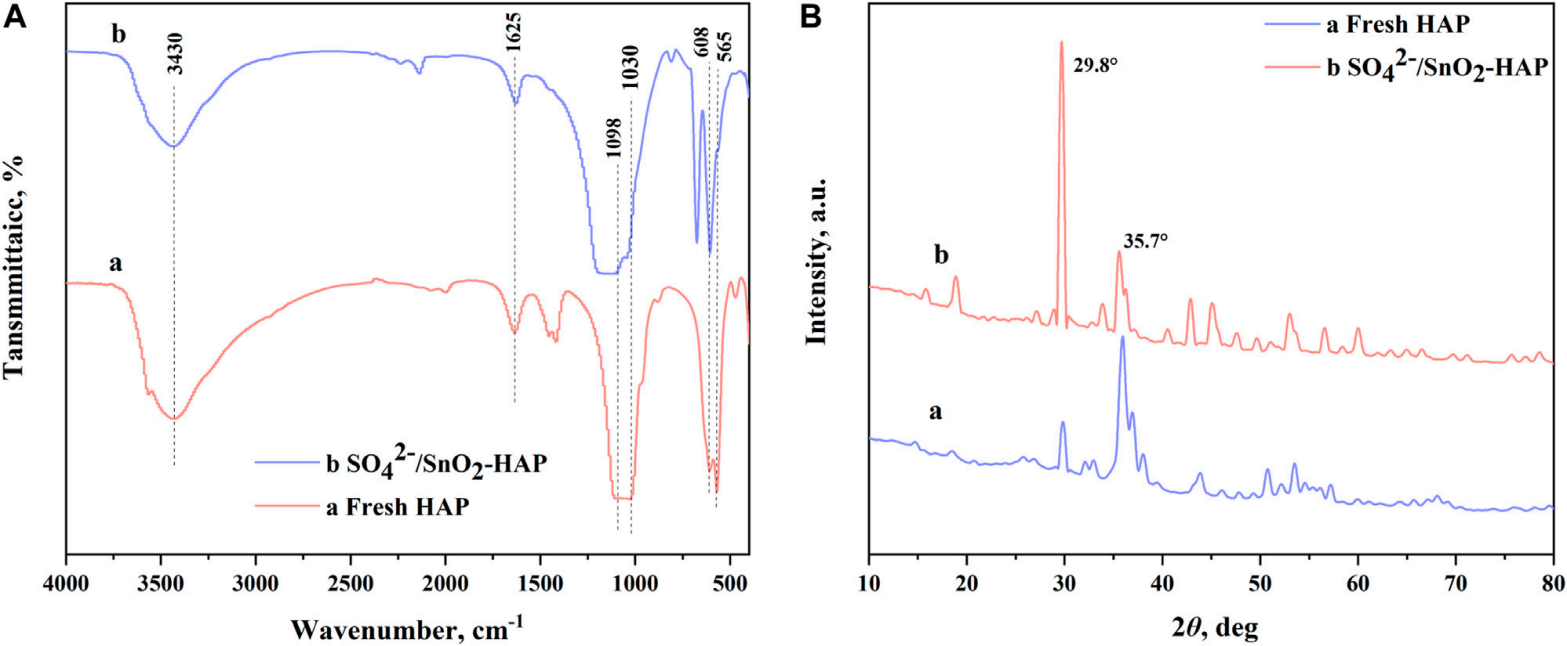
FT-IR **(A)** and XRD **(B)** of HAP and SO_4_^2−^/SnO_2_–HAP.

Using hydroxyapatite (HAP) as support, a sulfonated HAP-based heterogeneous catalyst was prepared for transforming CC into FF. Relative to carrier support HAP, SO_4_^2−^/SnO_2_–HAP had an enlarged surface area (51.9 m^2^/g) and decreased pore diameter (10.7 nm) (Table 1). Pore volumes of SO_4_^2-^/SnO_2_–HAP were increased to 0.14 cm^3^/g. SEM illustrated that SO_4_^2−^/SnO_2_–HAP had more voids (Figure 1), which caused the increased surface area and pore volume. FT-IR illustrated that the SO_4_^2−^/SnO_2_–HAP surface was distinct with a carrier HAP surface (Figure 2A). The bond of ∼ 3,430 cm^−1^ was related to Si–OH stretching. The bond of ∼ 1,098 cm^-1^, which was related to asymmetric stretching vibrations of Si–O–Si (Gong et al., 2019), increased after the synthesis of SO_4_^2−^/SnO_2_–HAP. The bond of ∼ 1,030 cm^−1^ was related to S=O (Peng et al., 2019), verifying the occurrence of SO_4_^2−^ on a catalyst. The bond of ∼ 790 cm^−1^ was related to Si–O–Si bending. XRD indicated that Sn ions modified the carrier HAP structure to some extent (Figure 2B). At 2*θ* = 20–30^o^, the SO_4_^2−^/SnO_2_–HAP intensity dropped compared to that of carrier HAP.

**TABLE 1:** BET results of the SO_4_^2−^/SnO_2_–HAP catalyst and the HAP carrier.

Sample	BET surface area, m^2^/g	Pore volume, cm^3^/g	Pore size, nm
SO_4_^2−^/SnO_2_–HAP	51.9	0.14	10.7
HAP	17.2	0.06	12.9

### 3.2 Co-catalysis of CC with organic acids and SO_4_^2−^/SnO_2_–HAP in water

**FIGURE 3:**
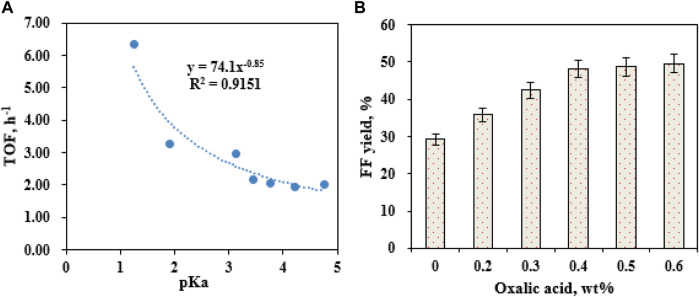
Effects of organic acids with different pKa values on the SO_4_^2−^/SnO_2_–HAP-catalyzed conversion of CC to FF after 10 min at 180°C **(A)**; effects of the oxalic acid dose (0.1–0.8 wt%) on FF generation after 10 min at 180°C **(B)**.

The catalytic system’s acidity might greatly affect FF generation (Marcotullio and de Jong, 2010). Maleic acid (pKa = 1.92), glyoxalate (pKa = 3.18), fumaric acid (pKa = 3.02), malic acid (pKa = 3.46), citric acid (pKa = 3.13), formic acid (pKa = 3.77), oxalic acid (pKa = 1.25), succinic acid (pKa = 4.21), acetic acid (pKa = 4.76), and propionic acid (pKa = 4.87) (0.5 wt%) were used to assist SO_4_^2-^/SnO_2_–HAP in the co-catalysis of CC into FF. The turnover frequency (TOF) of the catalytic reaction and pKa value of organic acids were well fitted into a curve equation (TOF = 74.1 × (pKa)^−0.85^; *R* ^2^ = 0.9151) (Figure 3A). In the acidic condition, a lower pKa value of organic acids favored FF formation with a higher TOF (Figure 3A). H^+^ in the lower pKa value of carboxylic acids was easier to dissociate into water. An increased acidity of the catalytic system would enhance the hydrolysis of hemicelluloses in lignocellulose into pentoses (e.g., *D*-xylose) and accelerate their dehydration to form FF (Lee and Wu, 2021). The highest TOF reached 6.33 h^−1^ *via* the co-catalysis of CC using oxalic acid (pKa = 1.25) and SO_4_^2−^/SnO_2_–HAP. Different dosages of oxalic acid (0.1–0.8 wt%) were separately supplemented to the catalytic system for assisting SO_4_^2−^/SnO_2_–HAP catalysis of CC within 10 min at 180°C (Figure 3B). Upon raising the oxalic acid dose from 0.1 to 0.4 wt%, FF yields increased from 24.6% to 48.2%. Over 0.4 wt%, FF yields had no significant change. Hence, 0.4 wt% of oxalic acid was utilized as a suitable additive to assist the SO_4_^2−^/SnO_2_–HAP catalysis of CC into FF.

### 3.3 Optimization of transforming CC into FF in DES EaCl:Gly–water

In the aqueous catalytic system, the low FF solubility might restrict FF generation, resulting in a decreased FF production (Li et al., 2021). DESs might be used as promising solvents for enhancing FF yields due to their unique properties, which would facilitate the generation of FF and confine undesired FF degradation or cross-polymerization (Pan et al., 2022). Using CC as a feedstock for producing FF, four factors including DES dosage, catalyst dose, performance temperature, and reaction time were examined for the effect of FF formation in DES EaCl:Gly–water.

**FIGURE 4:**
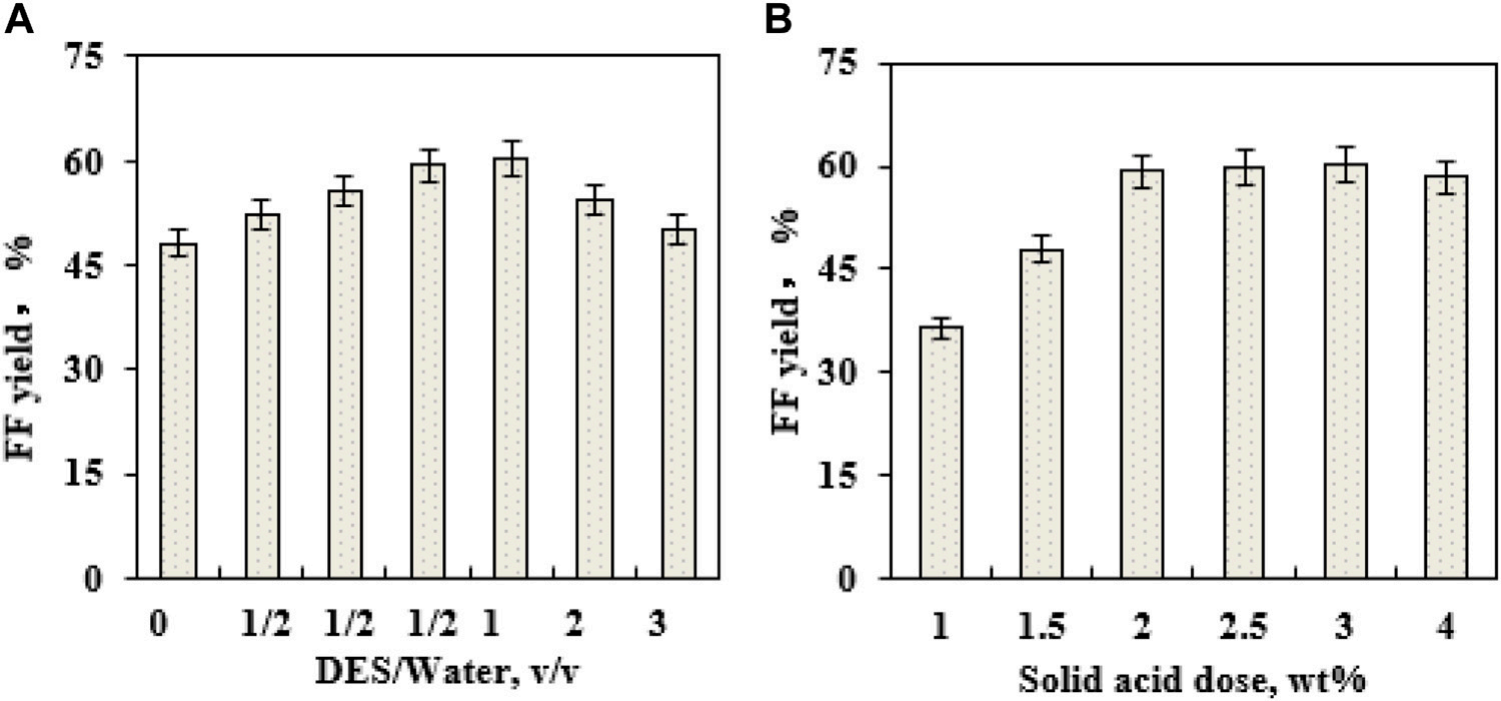
Effects of DES EaCl:Gly loading on FF generation (180°C; 10 min) **(A)**; effects of the SO_4_^2−^/SnO_2_–HAP dose on FF generation (180°C; 10 min) **(B)**.

When the ratio of DES–water is changed, the formed DES−water media might influence the production of FF (Pan et al., 2022). To examine EaCl:Gly dosage on FF generation, four volumetric ratios of EaCl:Gly–water (0:1, 1:3, 1:2, 1:1, 2:1, and 3:1; v/v) were individually utilized as catalytic reaction media (180°C) (Figure 4A). By raising the volumetric ratio of EaCl:Gly–water from 0:1 (EaCl:Gly, 0 vol%) to 1:2 (EaCl:Gly, 33.3 vol%), FF yields increased from 48.2% to 59.3%. Upon increasing the EaCl:Gly–water volumetric ratio from 1:2 to 1:1, FF yields increased slightly. Compared to the aqueous phase system, the merit of the DES–water system is that FF might be extracted into EaCl:Gly *in situ* quickly. Thus, not only the generation of by-products is confined but also the tedious performance steps for the extraction of FF are also saved. Over 1:1 (v/v), FF yields decreased from 59.3% to 50.1% in EaCl:Gly–water. In view of FF yields and EaCl:Gly dosage, the appropriate EaCl:Gly–water volumetric ratio was chosen as 1:2 (EaCl:Gly, 33.3 vol%). As the EaCl:Gly dose increased in EaCl:Gly–water, excessive solvent loading would reduce the contact opportunity of lignocellulose to catalysts and cause the reduction of FF yields (Li et al., 2021).

**FIGURE 5:**
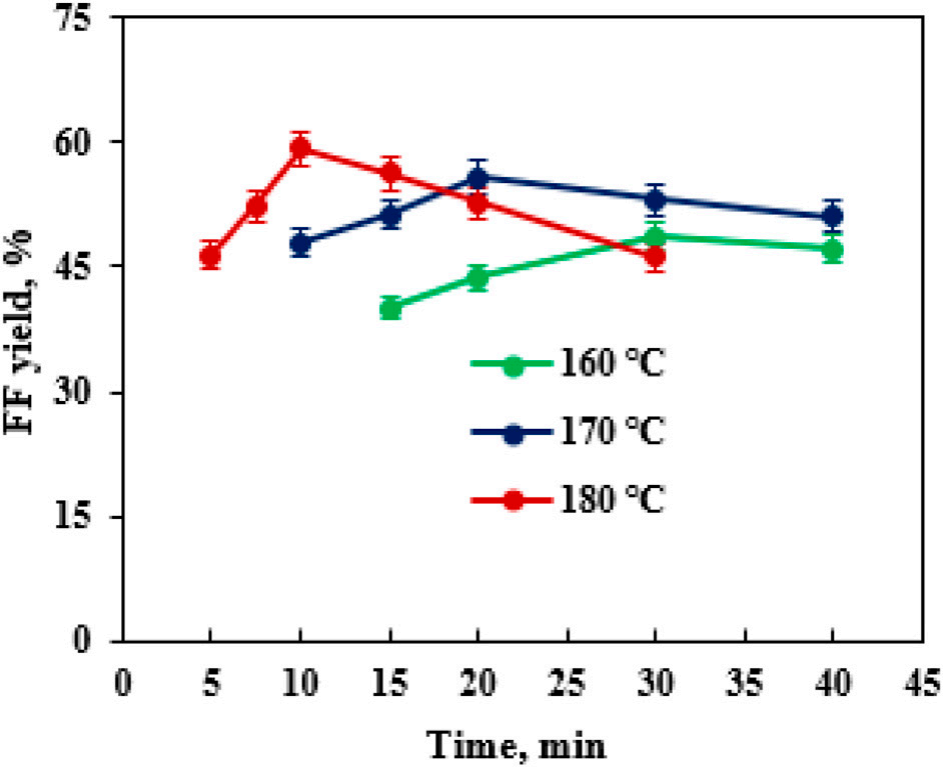
Effects of the performance temperature and time on FF generation.

During the conversion of biomass into FF with solid acids, catalyst loading had a profound influence on FF production (Peng et al., 2019). It was observed that the loading of SO_4_^2−^/SnO_2_–HAP (1–4 wt%) had a crucial effect on FF formation in EaCl:Gly–water (1:2, v/v) by supplementary oxalic acid (0.4 wt%) (Figure 4B). As the SO_4_^2−^/SnO_2_–HAP dosage increased from 1 to 2 wt%, FF yields improved from 36.4% to 59.3%, and when SO_4_^2−^/SnO_2_–HAP loading exceeded 2.0 wt%, FF yields showed no significant change. Thus, the optimal SO_4_^2−^/SnO_2_–HAP dosage was 2.0 wt%. The performance temperature and reaction time were pronounced as vital parameters that affected the production of FF (Zhang et al., 2019). After the transformation of CC was conducted for 5–40 min in a reactor (160–180°C) containing 40 mL EaCl:Gly–water (1:2, v/v), it was found that the highest FF yield (59.3%) was obtained at 180°C after 10 min *via* co-catalysis with SO_4_^2−^/SnO_2_–HAP (2.0 wt%) and oxalic acid (0.4 wt%) (Figure 5). By increasing the reaction time from 5 to 10 min, the FAL yield was increased significantly. Over 10 min, the FAL yield dropped gradually. Sn–sepiolite (3.0 wt%) catalyzed rice straw to FF (42% yield) in water after 20 min at 170°C (Peng et al., 2019). Sn–vermiculite (4.0 wt%) converted reed to FF (55.0 mM; 38.4% yield) in water at 170°C after 20 min (Zhu et al., 2020). Evidently, SO_4_^2−^/SnO_2_–HAP could be utilized to catalyze CC into FF with a high yield (59.3%) in EaCl:Gly–water (1:2, v/v) containing oxalic acid (0.4 wt%).

In the EaCl:Gly–water system, lignocellulosic biomass was catalyzed to FF and its derivatives by synergetic catalysis with SO_4_^2−^/SnO_2_–HAP and oxalic acid. Water molecules could break the complicated matrix of cellulose–lignin–hemicellulose, which would facilitate the promotion of the dissolution of hemicellulose (Lam et al., 2012). Carboxylic acids as Brönsted catalysts, which are also used as components of DESs, had a good biomass pretreatment ability (Yin et al., 2021). DES EaCl:Gly, which has good biomass pretreatment, could reduce the degradation of hemicellulose that enhanced the selective conversion of hemicellulose (Zhang et al., 2014). The xylan-derived *D*-xylose was dehydrated and opened in the presence of Cl^−^ and finally closed to FF (Marcotullio and de Jong, 2010). FF could also be obtained *via* the transformation of xylulose derived from isomerized *D*-xylose (Choudhary et al., 2011; Li et al., 2014). Glucan-derived glucose was isomerized to fructose, which would be further dehydrated to form 5-HMF and then hydrolyzed to produce HCOOH and levulinic acid (Wang et al., 2017).

### 3.4 Reuse of SO_4_^2−^/SnO_2_–HAP

**FIGURE 6:**
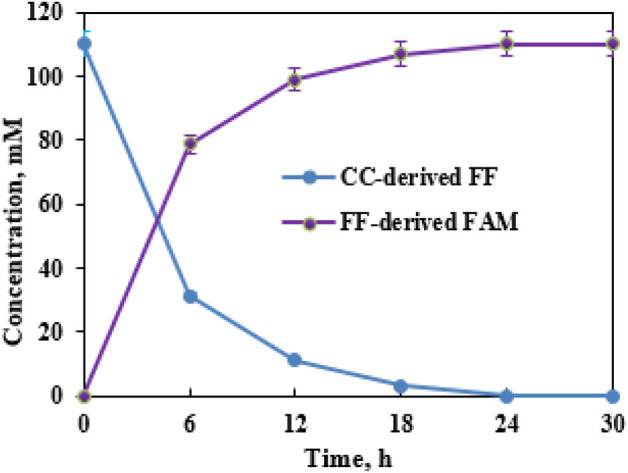
Bioamination of the CC-derived FF into FAM in EaCl:Gly–water (1:2, v/v; pH 7.5) at 35°C.

Reusability is a key indicator in assessing the performance of a heterogeneous catalyst (Gong et al., 2019). In this work, the catalyst SO_4_^2−^/SnO_2_–HAP was reused for eight cycles. After each catalytic cycle in EaCl:Gly–water (1:2, v/v), the mixture of biomass residue and SO_4_^2-^/SnO_2_–HAP was isolated by filtration and further washed with distilled water three times. Then, this solid mixture was burned in an oven to remove biomass residue. The remaining solid was further sulfonated before starting the next batch. The activity of SO_4_^2−^/SnO_2_–HAP was maintained during four consecutive tests (41.7%–59.3%) (Figure 6). FF yields decreased gradually after each batch. From the first to the fourth run, FF yields dropped from 59.3% to 51.6%. From the fifth to the sixth run, a significant decrease in FF yields (41.7%–47.8%) was obtained. After Sn–sepiolite was recovered and reused for six runs, the FF yield decreased from 42% to 35% (Peng et al., 2019). Evidently, SO_4_^2-^/SnO_2_–HAP had good stability and reusability. An efficient recovery and reuse process could reduce the operation cost of FF production.

### 3.5 Valorization of biomass-derived FF to FAM in EaCl:Gly–water

DESs can be utilized as eco-friendly reaction solvents for chemocatalysis and biocatalysis (Ni et al., 2021). In 40 mL EaCl:Gly–water (1:2, v/v), a tandem conversion of biomass into FAM was attempted *via* a sequential acidified SO_4_^2−^/SnO_2_–HAP chemocatalyst and a CCZU-XLS160 cell biocatalyst. Through co-catalysis with SO_4_^2−^/SnO_2_–HAP (2.0 wt%) and oxalic acid (0.4 wt%), corncob (CC), sugarcane bagasse (SB), rice straw (RS), and reed leaf (RL) (3.0 g, 75 g/L) could be catalyzed into 114.9, 79.6, 74.5, and 50.8 mM FF at 180°C after 10 min (Table 2), respectively. FF yields were obtained as follows: *Yield*_(CC)_ = 59.3% > *Yield*_(SB)_ = 47.3% > *Yield*_(RS)_ = 45.2% > *Yield*_(RL)_ = 42.4%. Various types of agro- and forest-wastes could be used to produce FF (Guenic et al., 2015; Gong et al., 2019; Lee and Wu, 2021). In this work, different types of biomass samples with different contents of xylan were used as feedstock for the production of FF. Overall, CC was a good feedstock for FF production due to the high xylan content (34.1 wt%) and FF yield (59.3%).

**TABLE 2:** Examination of the chemoenzymatic conversion potency toward different biomasses in EaCl:Gly–water (1:2, v/v).

Biomass	Xylan content in biomass, wt%	FF, mM (yield, %)^a^	FAM productivity, g FAM/g xylan^b^
Corncob (CC)	34.1	114.9 (59.3)	0.43
Sugarcane bagasse (SB)	29.6	79.6 (47.3)	0.34
Rice straw (RS)	29.0	74.5 (45.2)	0.33
Reed leaf (RL)	21.1	50.8 (42.4)	0.31

^a^ Various biomass samples (7.5 wt%) were used as feedstocks for the production of FF, after 10 min at 180°C in EaCl:Gly–water (1:2, v/v) by co-catalysis with SO_4_^2−^/SnO_2_–HAP (2.0 wt%) and oxalic acid (0.4 wt%).

^b^ The formed FF was aminated to FAM by supplementary CCZU-XLS160 cells (0.050 g/mL) and NH_4_Cl (2 mol NH_4_Cl/mol FF) in EaCl:Gly–water (1:2, v/v; pH 7.5) at 35°C for 24 h.

**FIGURE 7:**
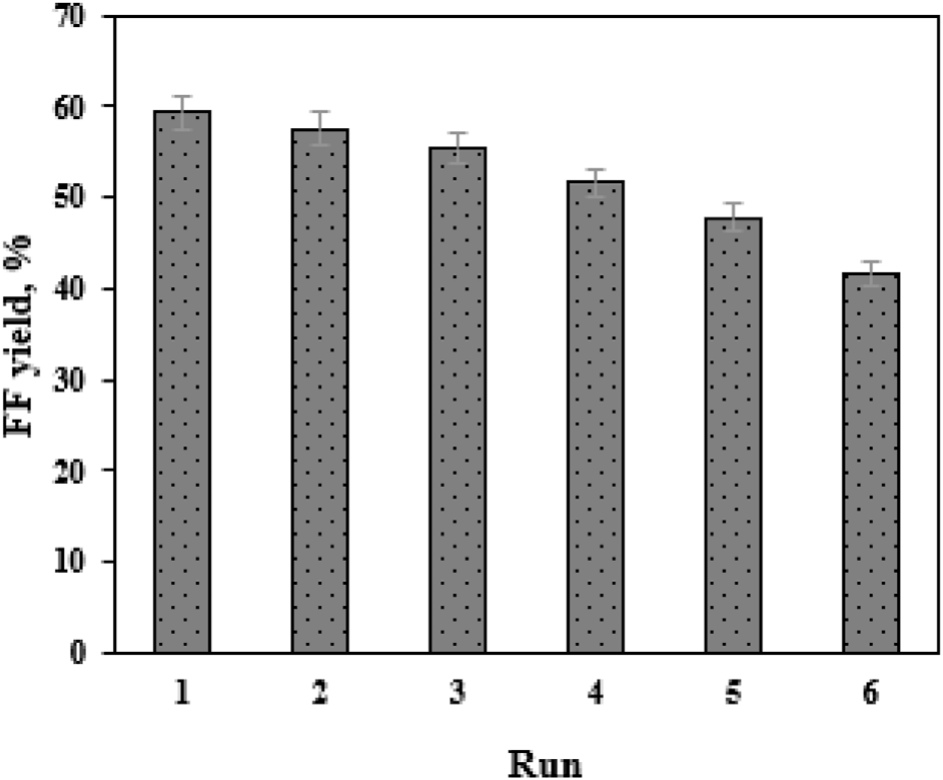
Reuse of SO_4_^2−^/SnO_2_–HAP in EaCl:Gly–water (1:2, v/v) at 180°C for 10 min.

The formed FF liquor, which was adjusted to pH 7.5, could be aminated into FAM by supplementary CCZU-XLS160 cells (0.050 g/mL) and NH_4_Cl (2 mol NH_4_Cl/mol FF) in EaCl:Gly–water (1:2, v/v). Time courses for the biotransformation of dilute CC-derived FF (110.0 mM) were monitored. By increasing the bioreaction time from 0 to 12 h, FF concentrations decreased quickly (Figure 7). After biological amination for 6 and 12 h, FAM concentrations reached 78.7 and 99.0 mM, respectively. When biological amination was carried out for 24 h, FF was fully transformed into FAM, achieving the productivity of 0.43 g FAM per g xylan. After biological transformations of SB-, RS-, and RL-derived FF for 24 h, the yields of FAM reached >99%, with the productivity of 0.31–0.34 g FAM per g xylan (Table 2). FF could be prepared from xylan in biomass *via* hydrolysis, dehydration, and cyclization reaction (Lee and Wu, 2021; Gong et al., 2022). Hence, the titer of FF derived from lignocellulosic biomass was mainly based on the xylan content in biomass. The biomass-derived FF could be wholly aminated to FAM by CCZU-XLS160 cells. It was observed that the generated FAM titer was largely determined by the xylan content of biomass in the tandem reaction with acidified SO_4_^2−^/SnO_2_–HAP and CCZU-XLS160 cells. Hence, CC could produce high productivity of FAM *via* a chemoenzymatic approach in EaCl:Gly–water (1:2, v/v).

Lignocellulosic biomass is an available, inexpensive, and renewable source, which has been widely used for the production of value-added chemicals (Mika et al., 2018; Lee and Wu, 2021). FF is a key bio-based chemical. One of its common preparation methods is conducted *via* catalysis by using metal salts, mineral acids, and organic acids as homogeneous catalysts. Recently, heterogeneous catalysts are regarded as environmentally friendly catalysts, which can be easily recovered and reused as compared to some homogeneous ones (Gong et al., 2019). It is known that the establishment of an efficient valorization process would achieve high concentrations of FF (Weingarten et al., 2010; Metkar et al., 2015). DESs have gained considerable interest as reaction solvents for FF production due to their low toxicity, good reusability, high thermostability, and ease of preparation (Xu et al., 2020). In this work, biomass samples (7.5 wt%) were utilized as feedstocks for preparing FF after 10 min in EaCl:Gly–water (1:2, v/v; 180°C) by co-catalysis with SO_4_^2−^/SnO_2_–HAP (2.0 wt%) and oxalic acid (0.4 wt%). Furthermore, biological amination of the prepared FF with whole-cell of CCZU-XLS160 could be used for the efficient production of FAM under an ambient performance condition. Tandem catalysis by bridging chemocatalysis and biocatalysis was constructed for the efficient and sustainable valorization of biomass into FAM. In the future, it is of great interest to establish a cost-effective catalytic process to obtain high FF concentration and thus further improve FAM productivity with high ω-transaminase activity.

## 4 Conclusion

To summarize, the establishment of an efficient chemoenzymatic approach for converting abundant, cheap, and renewable lignocellulosic biomass into value-added furans can be used as a sustainable strategy in the biorefinery process. In an eco-friendly reaction system, various types of lignocellulosic biomasses can be converted to FAM *via* sequential catalyses with solid acid catalysts and transaminase biocatalysts.

Using hydroxyapatite (HAP) as a carrier, solid acid catalyst SO_4_^2−^/SnO_2_–HAP was prepared for catalyzing xylan-rich biomass in this work. Organic acids were used to assist newly synthesized SO_4_^2−^/SnO_2_–HAP for FF production. It was found that oxalic acid (pKa = 1.25; 0.4 wt%) plus SO_4_^2−^/SnO_2_–HAP (2.0 wt%) gave the highest FF yield in EaCl:Gly–water (1:2, v/v) at 180°C after 10 min. SO_4_^2−^/SnO_2_–HAP had a good reusability performance, which could be reused for six runs.

FF liquors, which were obtained from different biomasses by co-catalyses with oxalic acid and SO_4_^2−^/SnO_2_–HAP in EaCl:Gly–water, could be fully aminated into FAM with the yield >99% with CCZU-XLS160 cells within 24 h. This established chemoenzymatic conversion strategy could be utilized in the valorization of renewable lignocellulosic biomass to valuable furan-based compounds in the eco-friendly EaCl:Gly–water system.

## Data Availability

The original contributions presented in the study are included in the article/Supplementary Material; further inquiries can be directed to the corresponding authors.
